# From pathogenesis to clinical application: insights into exosomes as transfer vectors in cancer

**DOI:** 10.1186/s13046-016-0429-5

**Published:** 2016-09-29

**Authors:** Wenting Xu, Zhen Yang, Nonghua Lu

**Affiliations:** Department of Gastroenterology, The First Affiliated Hospital of Nanchang University, 17 YongWaizheng Street, Nanchang, Jiangxi 330006 China

**Keywords:** Exosomes, Cancer, Transfer vectors

## Abstract

Exosomes are nanoscale extracellular membrane vesicles that are created by the fusion of an intracellular multivesicular body with the cell membrane. They are widely distributed in serum, urine, saliva and other biological fluids. As important transfer vectors for intercellular communication and genetic material, exosomes can stimulate target cells directly via receptor-mediated interactions or via the transfer of various bioactive molecules, such as cell membrane receptors, proteins, mRNAs and microRNAs, thus exerting their biological functions. This review focuses on the biological characteristics of exosomes, as well as their role and underlying mechanisms of action in the evolution of tumor formation, metastasis, drug resistance and other malignant behaviors. Additionally, this review emphasizes the potential applications of exosomes in the treatment of tumors. Further research may provide new ideas and methods to establish effective, exosome-based strategies for the early diagnosis and treatment of tumors.

## Background

In 1981, when Trams et al. studied vesicles in normal and tumor cells, they unexpectedly dicovered another group of vesicle-like substance which were smaller than multivesicular under transmission electron microscope [1]. Then, in 1987, Johnstone et al. named this kind of membrane vesicles as “exosomes”, and meanwhile, they observed exosome formation during reticulocyte maturation and successfully isolated and purified exosomes from reticulocytes by centrifugation at 100,000 x g for 90 min for the first time [2]. In 1996, Raposo et al. [3] found that in human B lymphocytes, some membrane vesicles isolated by differential centrifugation possessed the ability to present antigens. They expressed abundant major histocompatibility complex (MHC) II molecules on their surface and could present antigens to T cells, leading to T cell activation. Then, researchers found that except for within living cells, exosomes can also be detected in vitro in the cultures of different cell types, such as dendritic cells (DCs), epithelial cells, platelets, mesenchymal stem cells (MSCs), and tumor cells [4–8]. Exosomes are widely present in all body fluids, including saliva, blood, urine, cerebrospinal fluid, pleural effusion and ascites, suggesting that exosomes are not limited to the metabolic products of normal physiological and pathological conditions and that the secretion of exosomes is a universal cellular function [9]. Exosomes have been found to participate in many important physiological functions as the transmission medium for intercellular communication. Exosomes are involved in the regulation of the immune response, inflammation and tumor development [10–12]. In this review, we will conduct an in-depth discussion on not only the biological characteristics of exosomes and their relationship with tumors but also their potential clinical applications.

### Biological characteristics of exosomes

#### Generation of exosomes

**Fig. 1:**
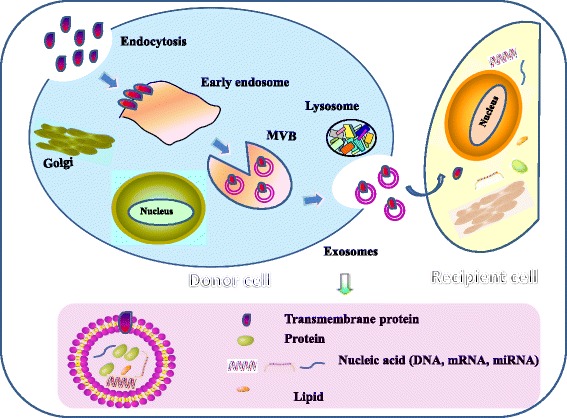
Biogenesis, release and upstake of exosomes

Exosomes are vesicle-like bodies that are secreted by cells and are 40 ~ 100 nm in diameter. As viewed by electron microscopy, exosomes are encompassed by a bilayer of phospholipid molecules, are cup- or plate-like in shape, and are usually enriched in a 1.13 ~ 1.19 g/ml sucrose density gradient solution [13]. Exosome synthesis and secretion involves a series of complex biological processes. First, a particular part of the cell membrane retracts, buds and forms an early endosome. Then, under the regulation of endocytosis-associated proteins and lipid raft complexes, early endosomes transform into late endosomes contained by intraluminal vesicles, i.e., multivesicular bodies (MVBs). At the same time, during the process of Ca^2+^-dependent ubiquitination and nucleic acid separation, some cytoplasmic proteins and nucleic acids become localized in MVBs [14, 15]. Finally, the MVBs that are not degraded by lysosomes will integrate and dock with the cell membrane with the participation of the Ras superfamily GTPase Rab; then, the MVBs release their contents into the extracellular space, creating exosomes [15]. In this process, some factors, such as platelet activation, radical pressure, decreased membrane cholesterol content and increased intracellular calcium levels, can increase the number of exosomes produced (Fig. 1).

### Molecular composition of exosomes

As potential biological material transporters, exosomes are usually composed of a series of biomolecules, including proteins, short-chain peptides, lipids, and fragments of DNA, mRNA, and microRNA (miRNA). The components of exosomes are closely related to the source and pathophysiological state of the secretory cells [16].

Based on traditional methods, such as SDS-PAGE and proteomic analysis, the proteins of exosomes have been mainly divided into two types, one of which includes common proteins distributed in every exosome, such as transmembrane transport and integration-related proteins (e.g., G protein, annexin, flotillin), tetraspanins (CD9, CD63, CD81, CD82) and heat shock proteins (Hsp70, Hsp90) [17, 18]. In particular, CD9 and CD63 are often used as molecular markers of exosomes that can be identified from a variety of extracellular vesicle-like structures, such as MVBs or apoptotic bodies; however, exosome-specific proteins have yet to be discovered [19]. The other protein type includes proteins that exist in specific types of exosomes; for example, MHC and costimulatory CD80 or CD86 molecules are abundant in exosomes surface originating from DCs and B lymphocytes, and a number of tumor antigens are contained in tumor cell-derived exosomes [20].

In addition to proteins, exosomes are also rich in lipids. The lipid components of exosomes are different from those of the cell membrane. Compared to their parent cells, exosomes is consist of phosphatidylserine, double-unsaturated phosphatidylethanolamine, double-unsaturated phosphatidylcholine, sphingomyelin, ganglia glycoside GM3, and cholesterol [16]. This unique combination is beneficial not only for maintaining high exosome stability but also for facilitating uptake by recipient cells.

Furthermore, exosomes contain a variety of non-coding RNAs or fragments, including overlapping protein-coding region RNA transcripts, repeats, structural RNAs, fragments of transfer RNAs, short hairpin RNAs, Y RNAs and small interfering RNAs (siRNAs) [21]. RNAs isolated from exosomes are more enriched than those in parental cells, which further demonstrates the selectivity of RNA incorporation into exosomes [22–24]. Many studies have detected abnormal miRNA expression in exosomes isolated from the body fluids of patients with various types of cancer, suggesting that miRNAs have potential as diagnostic and prognostic biomarkers [25–45] (Table 1).

**Table 1:** Exosomal miRNAs isolated from body fluids of cancer patients as potential biomarkers

Cancer type	Biofluid	Relevant miRNA	Reference
Lung cancer	Plasma	miR-151a-5p, miR-30a-3p, miR-200b-5p, miR-629, miR-100, miR-154-3p	[26]
	Plasma	let-7f, miR-30e-3p, miR-223, miR-301	[27]
	Plasma	miR-17-3p, miR-21, miR-106a, miR-146, miR-155 miR-191, miR-192, miR-203, miR-205, miR-210, miR-212, miR-214	[28]
Nasopharyngeal carcinoma	Serum	miR-24-3p, miR-891a, miR-106a-5p, miR-20a-5p, miR-1908	[29]
Esophageal	Serum	miR-21	[30]
squamous cell carcinoma	Serum	miR-1246	[31]
Breast cancer	Serum	miR-200a, miR-200c, miR-205	[32]
	Serum	miR-21	[33]
	Serum	miR-101, 372, 373	[34]
Hepatocellular carcinoma	Serum	miR-21	[35]
Pancreatic cancer	Serum	miR-17-5p, miR-21	[36]
	Serum	miR-1246, miR-4644, miR-3976, miR-4306	[37]
Prostate cancer	Plasma, Serum,	miR-107, miR-141, miR-375, miR-574-3p	[38]
	Urine, Serum	miR-141	[39]
Glioblastoma	Serum	miR-320, miR-574-3p	[40]
	Cerebrospinal fluid	miR-21	[41]
Colorectal cancer	Serum	Let-7a, miR-1229, miR-1246, miR-150, miR-21, miR-223, miR-23a	[42]
	Serum	miR-17-92a cluster	[43]
Ovarian cancer	Serum	miR-21, miR-141, miR-200a, miR-200b, miR-200c, miR-203, miR-205, miR-214	[44]
Cervical cancer	Cervicovaginal lavage	miR-21, miR-146a	[45]

These components (e.g., proteins, mRNA and miRNA) can be selectively incorporated into the exosomes [46]. However, the exact molecular events that regulates the packaging of exosomes either via vesicle-mediated or non-vesicle-mediated mechanisms are largely unclear.

### Source and isolation of exosomes

A variety of cell types have been used to produce exosomes in cancer research. In particular, immature dendritic cells (imDCs) have become one of the most popular donor cells due to their unique surface proteins [47]. CD9 on imDC-derived exosomes can promote the fusion of exosomes with the membrane of target cells, thus increasing the delivery of drugs [48]. Furthermore, exosomes derived from imDCs lack immunostimulatory T cell surface markers, such as CD40, CD86, and MHC II, and thus have reduced immunogenicity [20]. Therefore, they can effectively induce defensive immune responses after being loaded with tumor antigens. A clinical trial on pancreatic cancer has confirmed the safety of imDC-derived exosomes [49]. However, the clinical application of imDC-derived exosomes is limited by small-scale production. MSCs are often used as exosome donor cells in glioma-related studies [50]. Due to their considerable production of exosomes, MSCs have potential as an effective source of exosomes for clinical applications. However, the anti-tumor effect of MSC-derived exosomes remains controversial [51, 52]. Therefore, the selection of exosome donor cells must take both tissue-specific targets and yield into consideration.

**Fig. 2:**
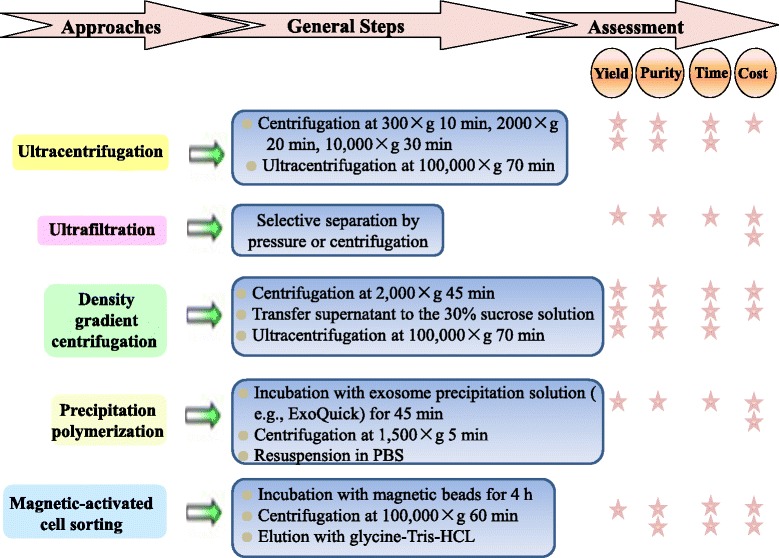
Approaches for exosome isolation. General procedures and assessment of the five commonly used approaches are shown

Considering the crucial role of exosomes in biological and clinical research, the methods by which exosomes are isolated from donor cells are very important. However, the unique size and structure of exosomes make their isolation and purification difficult. Frequently used methods include ultracentrifugation, ultrafiltration, density gradient centrifugation, precipitation polymerization, and magnetic-activated cell sorting. The differences of these five methods and assessments of their efficiency and cost are shown in Fig. 2 [53]. As each of these methods has advantages and disadvantages, so it is necessary to develop more efficient extraction methods.

### Tumor promotion role of exosomes

#### Exosomes and tumor proliferation

In prostate cancer, glioblastoma, breast cancer and other tumors, exosomes have been confirmed to be closely associated with tumor cell proliferation [54–56]. The underlying mechanisms can potentially be used as targets for cancer therapeutics.

Exosomes affect heterogeneous cells in the microenvironment by transferring a cancerous phenotype and inducing cells to undergo malignant transformation; thus, exosomes play an important role in cancer progression. For example, exosomes in the serum of breast cancer patients were injected into mice together with normal epithelial cells and ultimately induced the formation of tumors in mice, while exosomes derived from normal subjects did not have this capability [57]. Exosomes secreted from prostate cancer cells are able to transform adipose stem cells into cancer cells possibly induced by their cargo of proteins, mRNA and miRNAs [58]. Bone marrow-derived MSCs from patients with multiple myeloma contain high levels of cytokines and adhesion molecules, which can promote the growth of multiple myeloma cells and contribute to the infiltration of cancer cells into the surrounding stromal tissue [59]. Prostate cancer cell-derived exosomes can increase the proliferation of LNCaP and RWPE cells and inhibit aging; exosomes derived from another prostate cancer cell line, DU145, can increase the volume of xenograft tumors and enhance the serum levels of prostate specific antigen [54]. Thus, exosomes derived from malignant tumors have the ability to induce carcinogenesis or transformation in normal cells.

In addition, the increased number of tumor cells is closely related to elevated cell viability and decreased apoptosis and necrosis. The contents of exosomes are rich, and many of these materials can modulate the viability and apoptotic ability of recipient cells. Upon entering recipient cells, these materials activate proliferation-related gene products of the recipient cells, leading to changes in cellular behavior and an increase in tumor volume. Yang et al. [60] demonstrated that bladder cancer cell-derived exosomes have the ability to inhibit tumor cell apoptosis, thus promoting tumor progression. As such, inhibiting the formation and release of exosomes from bladder cancer cells could lead to new strategies for future bladder cancer treatments. Caspase-3 can induce cell apoptosis. Upon the inhibition of exosome release, caspase-3 will accumulate, leading to apoptosis [60]. Thus, tumor cells might inhibit caspase-3 accumulation by releasing exosomes containing caspase-3, thereby avoiding apoptosis.

Similarly, liver cancer cell-derived exosomes can transfer the highly conserved long non-coding RNA ucRNATUC339 to liver cancer cells, thereby promoting cell proliferation; the use of siRNA to inhibit the expression of TUC339 can reduce the proliferation of hepatocellular carcinoma (HCC) cells [61].

### Exosomes and tumor metastasis

The epithelial-to-mesenchymal transition (EMT) is a highly conserved biological process by which cells lose epithelial and gain mesenchymal characteristics, including changes in cell morphology and phenotype. This process is the initial step in tumor cell invasion and distant metastasis [62]. Josson et al. [63] demonstrated via morphological and biochemical findings that after stromal cell-derived exosomes transport of miRNAs into tumor cells, the tumor cells will undergo the EMT. Furthermore, many proteins found within exosomes are involved in the induction of the EMT, including transforming growth factor (TGF)-β, tumor necrosis factor-α, interleukin (IL)-6, tumor susceptibility gene 101, Akt, integrin-linked kinase 1, β-catenin, and matrix metalloproteinases [64, 65]. Functional studies have shown that exosomes use these bioactive components to induce the formation of a pro-metastatic phenotype in recipient cells. After the occurrence of the EMT, tumor cells have enhanced invasion and metastasis capabilities and a high level of malignancy, leading to a significantly worse prognosis [66].

In addition, exosomes may also mediate the transfer of metastasis-related substances between cells to confer those cells with the characteristic features necessary for distant metastasis [67]. CD44v6 overexpression in rat pancreatic carcinoma cells can induce a strong metastatic ability [68]. Jung et al. and Wang et al. [69, 70] reported that rat pancreatic cancer cell-derived exosomes loaded with CD44v6 might promote the formation of environments supporting tumor metastasis in rat lymph nodes and lungs and that CD44v6 is a potential biomarker for cells initiating pancreatic adenocarcinoma. Crossing the blood-brain barrier is a key step in the metastasis of tumor cells to the brain. Tominaga et al. [71] demonstrated that tumor cell-derived exosomes were involved in the destruction of the blood-brain barrier and thereby promoted tumor cell metastasis to the brain.

Moreover, exosomes released by tumor cells can also change the physiological status of both the surrounding tissue and distant cells, indirectly contributing to the growth and spread of cancer cells, e.g., by stimulating vascular permeability or adjusting the local environment of organs before metastasis occurs [72, 73]. For example, tumor cells can secrete exosomes with high levels of miR-122, which can inhibit glucose absorbance in non-cancerous cells before metastasis, thereby increasing the available energy supply at distant organ microniches (niches) and ultimately promoting disease development [74]. Thus, during the formation of pre-metastasis microenvironments, exosomes play a relatively obscure but crucial role. Exosomes released by highly invasive melanoma cells can remodel bone marrow precursor cells into vessel-like cells to increase the degree of primary cancer malignancy [75]. Pancreatic cancer cell-derived exosomes carrying macrophage migration inhibitory factor can induce Kupffer cells to secrete TGF-β, resulting in hepatic stellate cell activation and extracellular matrix remodeling. Such reprogramming of the microenvironment significantly promotes the infiltration of bone marrow-derived macrophages, which provide a favorable microniche for pancreatic cancer cells to metastasize to the liver [73].

Recent studies have found that tumor cell-derived exosomes can promote the metastasis of tumor cells through integrins. Additionally, the type of integrin can determine the metastatic direction of a malignant tumor, i.e., before primary tumor metastasis, released exosomes are first transferred to the target organ to prepare the environment for tumor cell colonization and facilitate metastasis [8]. Hoshino et al. [12] found that the integral proteins of exosomes derived from various types of tumor cells widely vary; integrins α6β4 and α6β1 play a key role in lung metastasis, and integrin αvβ5 plays a role in hepatic metastasis. After exosomes carrying integrins are absorbed, the adhesion ability of recipient cells will be enhanced, Src phosphorylation will be activated, and the expression of the proinflammatory gene S100 will be upregulated, thereby promoting tumor metastasis. These findings provide a new direction for the development of integrin-targeted anti-tumor drugs.

### Exosomes and angiogenesis

Tumor angiogenesis is the key to tumor growth, invasion and metastasis because new blood vessels can supply oxygen and other nutrients to tumor cells. Angiogenesis can usually be attributed to the pre-vascular angiogenic factors secreted by various cells in the microenvironment, such as vascular endothelial growth factor (VEGF), which stimulates neighboring endothelial cells to recruit mast cells and macrophages from the bone marrow [76]. However, exosomes are often involved in this process. Exosomes containing EGFRvIII that are released from tumor cells can induce autocrine VEGF signaling in endothelial cells, thus leading to VEGFR activation and subsequent neovascularization [77]. Proteins and/or mRNAs isolated from highly malignant glioblastoma multiforme (GBM) cells in patient plasma reflect the tumor-associated hypoxia status and invasion characteristics. In addition, under hypoxic conditions, exosomes released by GBM cells can induce the synthesis of factors that can stimulate the activation of the phosphoinositide 3-kinase/Akt signaling pathway or accelerate angiogenesis, among other tumor growth-related processes [78]. Similarly, tissue factor (TF) in colorectal cancer cells can directly connect the genetic state of cells (such as K-ras oncogene activation or the functional loss of the p53 tumor suppressor gene) with their angiogenic and proliferative abilities in vivo. Interestingly, exosomes containing TF can modulate angiogenesis and stimulate tumor growth in vivo, although they cannot promote the proliferation of cancer cells in vitro [79]. Furthermore, in the process of tumor angiogenesis, myofibroblasts in the tumor microenvironment also play an important role. Webber et al. [80] found that prostate cancer and mesothelioma cells release exosomes containing TGF-β, which acts on recipient cells and induces the differentiation of fibroblasts into myofibroblasts. Cho et al. [81] reported that after exosomes secreted by breast cancer cells were taken up by adipose tissue-derived MSCs, the SMAD signaling pathway was activated and myofibroblast differentiation began. Thus, exosomes released by tumor cells can recruit fibroblasts and induce fibroblast differentiation, leading to tumor angiogenesis.

### Exosomes and tumor immunity

Exosomes play different roles in the regulation of the immune system due to their different contents. Some exosomes have a strong inhibitory effect on the immune system, and the mechanism of action is involved with many aspects of the immune system.

Exosomes can promote the immunosuppressive effect of regulator T cells (Tregs) to inhibit the natural anti-tumor immune response. Studies have shown that exosomes derived from head and neck squamous cell carcinoma PCI-13 cells can induce Treg proliferation and differentiation and increase the expression of TGF-β [82]. Szajnik et al. [83] found that exosomes from the serum of patients with stage Шc ovarian cancer, fallopian tube cancer or primary peritoneal cancer also mediate the transformation of CD4^+^CD25^neg^ T cells into CD4^+^CD25^high^Foxp3^+^ Tregs, increase the expression of IL-10, TGF-1, FasL, cytotoxic T lymphocyte-associated antigen-4, perforin and granzyme B, and enhance STAT3 phosphorylation. In addition, Mrizak et al. [84] reported that exosomes carrying TGF-β protein could induce Treg amplification in nasopharyngeal carcinoma. Thus, as exosomes play an important role in Treg amplification, blocking exosome-mediated Treg amplification could serve as a tumor immunotherapy strategy.

Some exosomes can also pass the death signal via FasL, thus causing the apoptosis of T cells and exerting immunosuppressive effects. For example, exosomes derived from the ascites of patients with advanced ovarian cancer were found to have high expression levels of FasL, which inhibited the expression of CD3 and JAK3 and induced T cell apoptosis [85]. Colon cancer cell-derived exosomes expressing FasL can also cause the apoptosis of Fas-sensitive T cells, and this effect can be blocked by anti-FasL antibodies [86, 87].

In addition, Cereghetti et al. [88] reported that exosomes derived from HCC1806 breast cancer cells contained hsa-miR-146a, miR-29a and miR-21, as well as many other miRNAs related to lymphocyte development and function. These exosomes thus inhibited the development and activation of lymphocytes and were involved in the adaptive immune response. TW03 nasopharyngeal carcinoma cell-derived exosomes can inhibit the proliferation and differentiation of Th1 cells and Th17 cells, promote Treg differentiation, and suppress the immune response [26].

Exosomes derived from the TS/A tumor cells can down-regulate the expression of NKG2D and lead to the secretion of perforin via TGF-β, MHC I-related chain A/B and UL16 binding protein-3, thus inhibiting natural killer (NK) cell function as well as the innate immune response [89, 90].

Exosomes may also mediate M2 macrophage amplification locally at tumor sites. Meghan et al. [91] demonstrated that exosomes derived from myeloid-derived suppressor cells can cause M2 macrophage polarization by inhibiting the secretion of IL-12 by macrophages, which in turn inhibits T cell function.

### Exosomes and tumor resistance

Tumor cell resistance is the main reason for the failure of clinical tumor treatment, which limits the clinical application of chemotherapy drugs. Overcoming drug resistance has become essential for achieving clinical efficacy.

As a newly discovered means of intercellular communication, exosomes can transmit distinct signals (including those of cellular drug resistance activity) among cells via drug efflux, which is a potential mechanism related to tumor resistance. Early clinical studies have suggested that exosomes can mediate the transfer of resistance from docetaxel-resistant prostate cancer cells to non-resistant cells, which is accompanied by changes in cell proliferation and invasiveness [92]. Additionally, exosomes released by cisplatin-resistant ovarian cancer cells contained not only cisplatin but also the drug transport protein ATP-binding cassette, subfamily C, member 2 and ATPase copper transporting alpha and beta, the latter of which activate signaling pathways in recipient cells, eventually reducing the sensitivity of the originally drug-sensitive cancer cells [93]. In breast cancer cell lines, tumor cells are not only able to pass a key molecule that mediates multidrug resistance, i.e., P-glycoprotein (P-gp), conferring the recipient cells with multidrug resistance, but also can regulate the expression level of P-gp by passing miRNA in order to enhance congenital resistance [94–96].

Cross-talk between stromal cells and cancer cells is also important for driving the resistance of breast cancer cells to chemotherapy and radiation therapy. During exchanges among heterogeneous cells, exosomes will be transferred from stromal cells to breast cancer cells. The RNAs within the exosomes are mostly non-coding transcripts and transposable elements, which can stimulate pattern recognition receptors and roundabout guidance receptors, thus activating STAT-dependent signaling [97]. In this way, stromal cells can activate the Notch3 pathway in breast cancer cells; then, stromal cells can release exosomes to coordinate with Notch3, thus promoting the proliferation of those resistant cancer cells [98]. Therefore, in the development of tumor resistance, cancer cells and surrounding stromal cells in the microenvironment can have a significant and far-reaching impact.

### Exosomes and tumor therapy

#### Exosomes as transport carriers for targeted cancer therapeutics

As exosomes enable the exchange of information among tumor cells, the specific nucleic acids and proteins they carry can provide new targets for the treatment of cancer. Compared with conventional materials used for targeted biological therapies, exosomes have many advantages. 1) The large number of proteins and the genetic information contained within exosomes suggest that exosomes can be loaded with most biological substances. 2) Exosomes are widely distributed in various human body fluids, indicating a good tolerance in humans; therefore, exosomes carrying drugs could exhibit a relatively long circulating half-life in vivo and thus demonstrate improved efficacy. 3) Exosomes can pass through the cell membrane and deliver carried substances to target cells; for example, DC-derived exosomes can transfer MHC molecules to other DCs, regulating the immune response. 4) Exosomes exhibit unique directionality, and many studies indicate that the targeting function of exosomes is related to the cell source. 5) Exosomes can modify their membrane and enhance their cell-specific targeting function; for example, exosomes loaded with siRNA toward beta-secretase 1 (BACE1) could cross the blood-brain barrier and down-regulate mRNA and protein BACE1 expression by 60 % in brain neurons, astrocytes and oligodendrocytes. 6) Different from traditional synthetic liposomes, exosomes can carry drugs both in vivo and in vitro; in vivo, drugs can be loaded into cells by transfection to obtain targeted exosomes; in vitro, drugs can be loaded into purified exosomes by electroporation and lipid transfection [99, 100].

In recent years, a number of studies have shown that exosomes, as a natural source of nanoscale vesicles, can transfer exogenous RNAs (including siRNAs and miRNAs) by specifically targeting tissues or cells for the purpose of silencing genes and suppressing tumor growth in mice [101, 102]. Shtam et al. [103] revealed that siRNAs can be successfully transferred to targeted cells by means of exosomes for the purpose of gene therapy.

In addition, exosomes may also mediate mRNA/protein transfer among cells and could potentially be used to deliver therapeutic genes to tumor cells, replace dysfunctional genes, and induce either an immune response or tumor cell apoptosis [104]. Mizrak et al. [105] found that after exosomes enriched with cytosine deaminase-uracil phosphoribosyltransferase (CD-UPRT) mRNA/protein were injected into murine nerve sheath tumors, the exosomes could release encapsulated CD-UPRT mRNA/protein into the target cells. These exosomes coordinated with 5-fluorocytosine (5-FC) to treat murine schwannoma and could promote the transformation of 5-FC into 5-fluorodeoxyuridine monophosphate, significantly inducing tumor cell apoptosis and tumor regression. On the other hand, in the process of inducing bone marrow cell differentiation into DCs, cells were co-cultured overnight with ovalbumin (OVA) and α-galactose (αGC); the extracted exosomes were enriched with the protein and glycolipid antigens. Further results showed that exosomes loaded with αGC-OVA could activate invariant NK cells, overcome the effect of αGC, and enhance the tumor-specific adaptive immune response; these findings support the development of novel cancer immunotherapies [106]. A phase I clinical trial showed that exosomes extracted from heat-treated carcinoembryonic antigen (CEA) + tumor-derived LS-174 T cells were enriched in CEA, HSP70 and MHC I. Then, the exosomes were co-cultured with mononuclear cells that were extracted from the serum of CEA+ patients, and the resulting sensitized DCs were injected into patients to induce a strong CEA-specific T cell anti-tumor effect. The test results showed that 60 % of patients had a CEA-specific T cell response and that the treatment was well tolerated; however, the immunostimulation was limited and requires further optimization [107].

Exosomes can also be loaded with other types of materials, e.g., chemotherapy drugs, for therapeutic applications. Recent studies have found that imDC-derived exosomes can be modified by iRGD and electroporation to carry doxorubicin. These exosomes can target αv integrin via the co-expression of Lamp2b, which can eventually lead to the directional transfer of doxorubicin into breast cancer MDA-MB-231 cells, MCF-7 cells, murine melanoma B16-F10 cells and human HCC cells, e.g., HepG2, as these cells have high αv integrin expression levels [108]. Due to its low bioavailability and poor stability, the application of curcumin in cancer and other inflammatory diseases is limited; however, exosomes can improve the stability of this natural medicinal compound [109]. Studies have shown that via co-incubation, curcumin can self-assemble into the lipid bilayer of exosomes and thereby escape degradation, resulting in improved stability and bioavailability [110]. Recently, Hood et al. [111] found that replacing phosphate-buffered saline with trehalose pulse media during electroporation can enable the successful loading of exosomes with heavier cargo, such as superparamagnetic iron oxide nanoparticles. This work not only achieved the loading of exosomes with semi-synthetic nanoparticles but also highlighted the diagnostic potential of exosomes in magnetic resonance imaging [112].

### Exosomes in anti-tumor vaccines

Aside from loaded exosomes, unloaded tumor cell-derived exosomes can also be effective as prophylactic and therapeutic anti-cancer vaccines, and such vaccines have produced good anti-tumor effects in murine tumor models. Mice immunized with tumor cell-derived exosomes exhibited a tumor formation rate that was significantly lower than that of mice immunized with tumor cell lysates [113]. Numerous studies have shown that tumor cell-derived exosomes carrying large quantities of tumor-specific antigens can induce DCs to release exosomes. Such exosomes can be loaded with tumor-specific antigens, e.g., costimulatory molecules, to induce an anti-tumor immune response [114]. A previous clinical trail has confirmed the safety of this tumor immunotherapy. Morse et al. [115] demonstrated that the condition of some non-small-cell lung cancer patients stabilized after treatment with exosomes loaded with melanoma antigens. Research on liver cancer cell-derived exosomes has shown that exosomes carrying heat shock proteins can enhance tumor immunogenicity and the immune response of induced NK cells, which provides valuable clues for the development of efficient liver cancer vaccines.

### Specific removal of exosomes

Since tumor-derived exosomes play an important role in tumor development, blocking the highly efficient secretion of these exosomes, intercepting exosomes that are secreted into the peripheral circulation, and inhibiting the absorption of exosomes by recipient cells are potential exosome-specific therapeutic strategies [116]. Aethlon Medical specially designed a dialysis method called Aethlon ADAPT^TM^, which uses antibodies and affinity reagents to achieve the specific removal of tumor cell-derived exosomes [117].

## Conclusions and propects

**Fig. 3:**
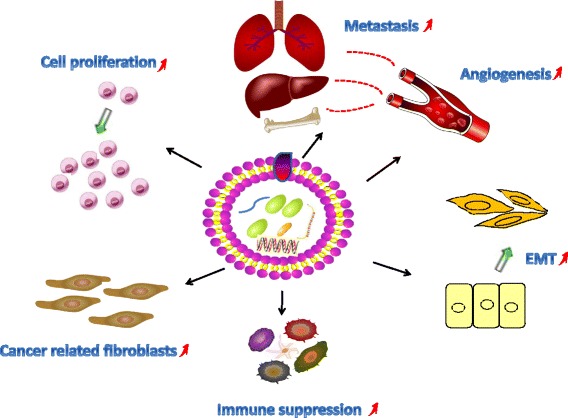
Underlying mechanisms by which exosomes induce formation, growth, metastasis, resistance in development of cancer

In recent years, the rapid development of the exosome field has attracted the attention of many scientists and clinicians. As a newly discovered means of intercellular information transfer, exosomes play an important role in tumorigenesis (Fig. 3) and have attractive prospects for application in the early diagnosis and treatment of tumors as well as in the assessment of prognoses. However, exosome research is still in an early stage, and many problems, such as the following examples, require further exploration before they can be resolved. 1) Confirming the contents of exosomes is critical for the safe production and effective synthesis of exosomes. However, there is still a lack of not only accurate and versatile methods for characterizing exosomes but also rapid, economical, efficient and standardized separation technologies; furthermore, there is a lack of information regarding the differences in the formation mechanisms, components and functions of exosomes in different conditions. 2) Exosomes can be used as a delivery system based on their unique characteristics, and they are superior to artificial carriers in many ways. However, considering the potential administration routes, dosages, and costs, the design principles for loaded exosome-based therapies require further improvement. 3) The regulation of mRNAs by exosomes enriched with tumor-promoting miRNAs is not fully understood. In addition, whether recipient cells exhibit selectivity when exosomes deliver functional proteins and RNAs remains to be determined. Clarifying and resolving these scientific issues and technical barriers may provide significant insights into the unique biological structures and functions of exosomes. In addition to influencing the development of future drug carriers, such insights could provide not only safe and convenient biomarkers for the early diagnosis of cancer but also targeted treatment strategies for individual biological therapies.

## Abbreviations

5-FC: 5-fluorocytosine
BACE1: Beta-secretase 1
CD-UPRT: Deaminase-uracil phosphoribosyltransferase
CEA: Carcinoembryonic antigen
DCs: Dendritic cells
EMT: Epithelial-to-mesenchymal transition
GBM: Glioblastoma multiforme
HCC: Hepatocellular carcinoma
IL: Interleukin
MHC: Major histocompatibility complex
miRNA: MicroRNA
MVBs: Multivesicular bodies
NK: Natural killer
OVA: Ovalbumin
P-gp: P-glycoprotein
siRNAs: Small interfering RNAs
TF: Tissue factor
TGF-β: Transforming growth factor-β
VEGF: Vascular endothelial growth factor
αGC: α-galactose.
